# 60 A Retrospective Review of High-Frequency Percussive Ventilation in Inhalation Injury

**DOI:** 10.1093/jbcr/irad045.034

**Published:** 2023-05-15

**Authors:** Emily Miller, Xu Zhang, Todd Huzar, John Harvin

**Affiliations:** McGovern Medical School at UTHealth, Houston, Texas; McGovern Medical School at UTHealth, Houston, Texas; McGovern Medical School at UT Health, Houston, Texas; UT Health Houston, Houston, Texas; McGovern Medical School at UTHealth, Houston, Texas; McGovern Medical School at UTHealth, Houston, Texas; McGovern Medical School at UT Health, Houston, Texas; UT Health Houston, Houston, Texas; McGovern Medical School at UTHealth, Houston, Texas; McGovern Medical School at UTHealth, Houston, Texas; McGovern Medical School at UT Health, Houston, Texas; UT Health Houston, Houston, Texas; McGovern Medical School at UTHealth, Houston, Texas; McGovern Medical School at UTHealth, Houston, Texas; McGovern Medical School at UT Health, Houston, Texas; UT Health Houston, Houston, Texas

## Abstract

**Introduction:**

Inhalation injury (II) is a major contributor to mortality in burn patients. These patients often require mechanical ventilation, but there is no clear consensus on optimal ventilatory strategy for this population. Previous studies in patients with acute respiratory distress syndrome (ARDS) demonstrated that high frequency percussive ventilation (HFPV) improved oxygenation and mobilization of airway secretions. However, HFPV in the context of II remains poorly studied. We hypothesized that for these patients, use of HFPV in addition to conventional mechanical ventilation (CMV) would reduce complications and decrease mortality compared to patients treated with CMV only.

**Methods:**

We conducted a retrospective chart review of 200 patients admitted to a burn center from February 2008 - January 2019 with burns and/or II and ventilated either with CMV alone or with CMV + HFPV. Patients who were under the age of 18 or who received mechanical ventilation for less one day were excluded. Information including patient demographics and comorbidities, percent total body surface area burned, ventilation method, length of stay, complications, and outcomes were collected. Two-sample comparisons were performed by using score test for proportions and Wilcoxon rank sum test for continuous variables with significance defined as p< 0.05.

**Results:**

Of 200 patients studied, 84 received CMV + HFPV and 116 received CMV alone. There was no difference in mortality between groups (CMV+HFPV 39.3% vs CMV 31.0%; p=0.23). There was also similar incidence of pneumonia (CMV+HFPV 53.6% vs CMV alone 63.8%; p=0.15), pneumothorax, ARDS, and sepsis. Patients on CMV + HFPV had a greater incidence of acute kidney injury (CMV+HFPV 60.7% versus CMV alone 43.1%; p=0.014) and reduced development of wound complications (CMV+HFPV 7.1% versus CMV alone 16.4%; p=0.05). In surviving patients (n=131), length of hospital stay was not significantly different between groups.

**Conclusions:**

These single-center results do not support the use of CMV + HFPV rather than CMV alone in patients with II. Further research in this area, including larger multi-center studies, is warranted.

**Applicability of Research to Practice:**

This is a hypothesis-generating study that adds to the currently limited understanding of best practice for managing patients with II.

